# Low‐risk MDS—A spotlight on precision medicine for *SF3B1‐*mutated patients

**DOI:** 10.1002/hem3.70103

**Published:** 2025-03-21

**Authors:** Shoshana Burke, Onima Chowdhury, Kevin Rouault‐Pierre

**Affiliations:** ^1^ Centre for Haemato‐Oncology Barts Cancer Institute, Queen Mary University of London London UK; ^2^ Oxford University Hospitals NHS Foundation Trust Oxford UK; ^3^ Molecular Haematology Unit, Weatherall institute of Molecular Medicine NHR, Biomedical Research Centre University of Oxford Oxford UK

## Abstract

A deep understanding of the biological mechanisms driving the pathogenesis of myelodysplastic neoplasms (MDS) is essential to develop comprehensive therapeutic approaches that will benefit patient's disease management and quality of life. In this review, we focus on MDS harboring mutations in the splicing factor *SF3B1*. Clones harboring this mutation arise from the most primitive hematopoietic compartment and expand throughout the entire myeloid lineage, exerting distinct effects at various stages of differentiation. Supportive care, particularly managing anemia, remains essential in *SF3B1*‐mutated MDS. While *SF3B1* mutations are frequently linked with ring sideroblasts and iron overload due to impaired erythropoiesis, the current therapeutic landscape fails to adequately address the underlying disease biology, particularly in transfusion‐dependent patients, where further iron overload contributes to increased morbidity and mortality. Novel agents such as Luspatercept and Imetelstat have shown promise, but their availability remains restricted and their long‐term efficacy is to be investigated. Spliceosome modulators have failed to deliver and inhibitors of inflammatory pathways, including TLR and NF‐κB inhibitors, are still under investigation. This scarcity of effective and disease‐modifying therapies highlights the unmet need for new approaches tailored to the molecular and genetic abnormalities in *SF3B1*‐mutated MDS. Emerging strategies targeting metabolic mis‐splicing (e.g., *COASY*) with vitamin B5, pyruvate kinase activators, and inhibitors of oncogenic pathways like MYC and BCL‐2 represent potential future avenues for treatment, but their clinical utility remains to be fully explored. The current limitations in treatment underscore the urgency of developing novel, more effective therapies for patients with *SF3B1*‐mutated MDS.

## INTRODUCTION

Myelodysplastic neoplasms (MDS) arise due to the acquisition of somatic mutations in hematopoietic bone marrow stem cells, resulting in ineffective hematopoiesis with subsequent cytopenias and a propensity for transformation to acute myeloid leukemia.^1^, ^2^, ^3^, ^4^ MDS is typically detected in older patients with a median age of 74 years.^5^ Most patients present with nonspecific symptoms such as fatigue and breathlessness, with anemia being the most common cytopenia detected in 80%–85% of all MDS patients.^6^, ^7^

MDS has two typical natural histories, which are delineated by the stratification of patients on their likelihood of progressing to AML or the likelihood of reduced overall survival. Risk stratification has evolved over the past 20 years from the IPPS score^8^ through to the revised IPPS (IPSS‐R), to the incorporation of valuable molecular data leading most recently to the IPSS‐M.^9^ Common to all scoring systems, higher‐risk patients transform to acute myeloid leukemia^3^ more rapidly, requiring disease modifying therapy and allogeneic stem cell transplantation if appropriate, with a median survival of around only 15 months without transplantation.^10^, ^11^, ^12^, ^13^, ^14^ However, most patients with MDS have lower‐risk disease, principally suffering from cytopenias, most commonly symptomatic anemia, which is a major unmet need. Patients develop consequences of symptomatic anemia, including a poor quality of life, increased cardiovascular complications, and a reduced life expectancy.^15^, ^16^ Currently, the majority of these patients will become reliant on red cell transfusions. While the term “low‐risk” may lead to underestimation of the condition's impact,^17^ these are burdensome for the patient and healthcare services. They also carry significant long‐term complications including organ damage due to iron overload.^18^ Ameliorating anemia with targeted therapies has the potential for huge benefits at the individual and population level but such novel drugs should be precisely targeted toward the subset of patients who are most likely to benefit.

One such subgroup of MDS patients is those now diagnosed with *SF3B1*‐MDS. This was formally classified by the WHO in 2022 as a distinct entity, due to homogeneity within *SF3B1‐*mutated patients with erythroid dysplasia, ring sideroblasts, severe anemia, and a relatively good prognosis.^19^ Correlation of certain genetic alterations can identify groups of patients who will selectively benefit from certain therapies. In an era of increasingly stretched healthcare resources, precision medicine is essential to enable the appropriate patients to receive novel therapies. In this review, we will focus on clinically relevant biology and the management of patients with *SF3B1‐*mutated MDS.

## CLASSIFICATION

In 2022 as part of the 5th edition of the World Health Organization (WHO) Classification of Hematolymphoid Tumours, the classification of MDS was revised. MDS was renamed as Myelodysplastic Neoplasms (although the acronym MDS remains), and the classification now incorporates critical genetic aspects of the disease and links closely with our revised knowledge of prognosis based on genetics.^20^, ^21^, ^22^ At the same time, the International Consensus Classification (ICC) of MDS was written, also emphasizing the molecular and genetic basis of MDS.^23^ Both classifications define *SF3B1‐*mutated MDS as a distinct subtype, with a unique disease course, which is mirrored in prognosis and therapeutic options (Table 1).^24^, ^25^ The novel entity of “MDS/AML” was also added in the ICC, defined as 10%–19% blasts in the peripheral blood or bone marrow without AML‐defining genetic abnormalities. The WHO renamed “MDS with increased blasts 2” (MDS‐IB2) and retained its 20% blast AML cut‐off. Although this review is focused on spliceosome mutations, these changes in nomenclature have consequences when it comes to enrolling patients in clinical trials, interpretation of future trial data, and treatment consequences for patients. To validate and digest the two classification systems, the International Committee for MDS (icMDS) applied both WHO and ICC classifications to a cohort of 2231 molecularly annotated MDS patients, and their clinic outcome was noted. One of the major points of agreement was that the genetically defined categories, *SF3B1*, (del)5q, and *TP53*, had distinct clinical courses and outcomes,^26^ highlighting the consistency and importance of careful molecular annotation (Table 1).

**Table 1:** Benchmarking of the different systems of MDS classification.

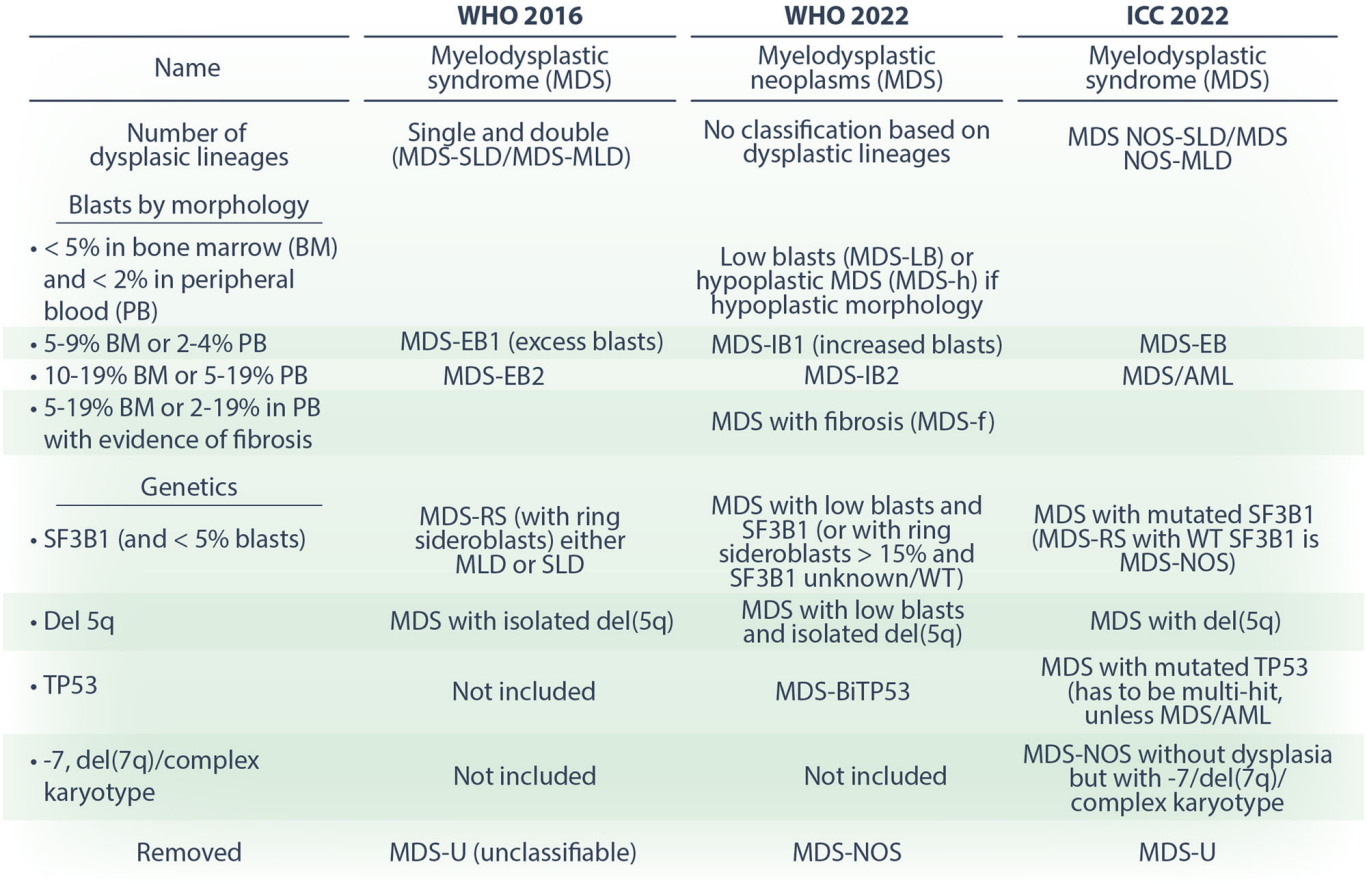

Abbreviations: EB, excess blasts; IB, increased blasts; ICC, International Consensus Classification; MDS, myelodysplastic neoplasms/syndromes; MDS‐f, MDS with fibrosis; MDS‐h, hypoplastic MDS; MDS‐LB, MDS with low blasts; MLD, multilineage dysplasia; NOS, not otherwise specified; SLD, single lineage dysplasia; RS, ring sideroblasts; WHO, World Health Organization; WT, wild type.

While undertreating patients who should have intensive therapy poses a real risk, so does overtreating patients who have a low risk of transforming from MDS to AML and a low risk of dying from the disease with unnecessary and potentially toxic drugs. In 1997, the International Prognostic Scoring System (IPSS)^27^ was published and was the first MDS scoring system to categorize patients by their risk of progression to AML and their risk of dying. This was revised (IPSS‐R) in 2012^8^ These early prognostic models include the number and severity of cytopenias, blasts in bone marrow, and some cytogenetic abnormalities. However, only 40%–50% of patients with MDS have detectable chromosomal copy number alterations, most commonly involving chromosomes 5, 7, 8, and 20.^28^ As was reflected in the diagnostic classification systems, recurrent genetic mutations have also been shown to have strong prognostic significance^29^, ^30^, ^31^ and have been integrated into several risk‐scoring systems.^9^, ^32^, ^33^ The most recent is the IPSS‐M.^9^, ^34^ For the IPSS‐M, pretreatment samples were taken from 2957 patients with potential MDS, and 152 genetic mutations were profiled. These data were added to and correlated with clinical variables and weighted using a Cox multivariable model, which was adjusted for confounders and mapped against leukemia‐free survival, leukemic transformation, and overall survival and validated in an external cohort of 754 Japanese MDS patients. The final calculation considers hematologic parameters, cytogenetic abnormalities, and somatic mutations of 31 genes.^9^

Although the availability of molecular testing and the cost remains an inequality across the world, the close link between molecular markers and prognosis, especially *SF3B1*‐mutant, del(5q), and *TP53*, makes assessment for these mutations essential.^34^ While most patients who morphologically have ring‐sideroblasts on bone marrow examination have *SF3B1* mutations, outcomes of *SF3B1* wild‐type patients with ring‐sideroblasts were more like MDS low blasts than *SF3B1‐*mutated disease, highlighting the importance of both morphological assessment and molecular testing.^35^

The optimal use of these prognostic systems to guide treatment remains an open question. Specifically, should patients with low‐risk IPSS‐R but an adverse molecular profile be managed as low‐risk, or should they be considered for intensive chemotherapy and/or bone marrow transplant? It is crucial to remember that a significant cause of morbidity in low‐risk MDS patients arises from co‐morbidities, which are exacerbated or made more challenging to treat by cytopenia. This aspect is not captured in the prognostic scoring system, underscoring that prognostic scores are one of many factors used to guide clinical decision‐making.

## MDS AND *SF3B1* BIOLOGY

**Figure 1:**
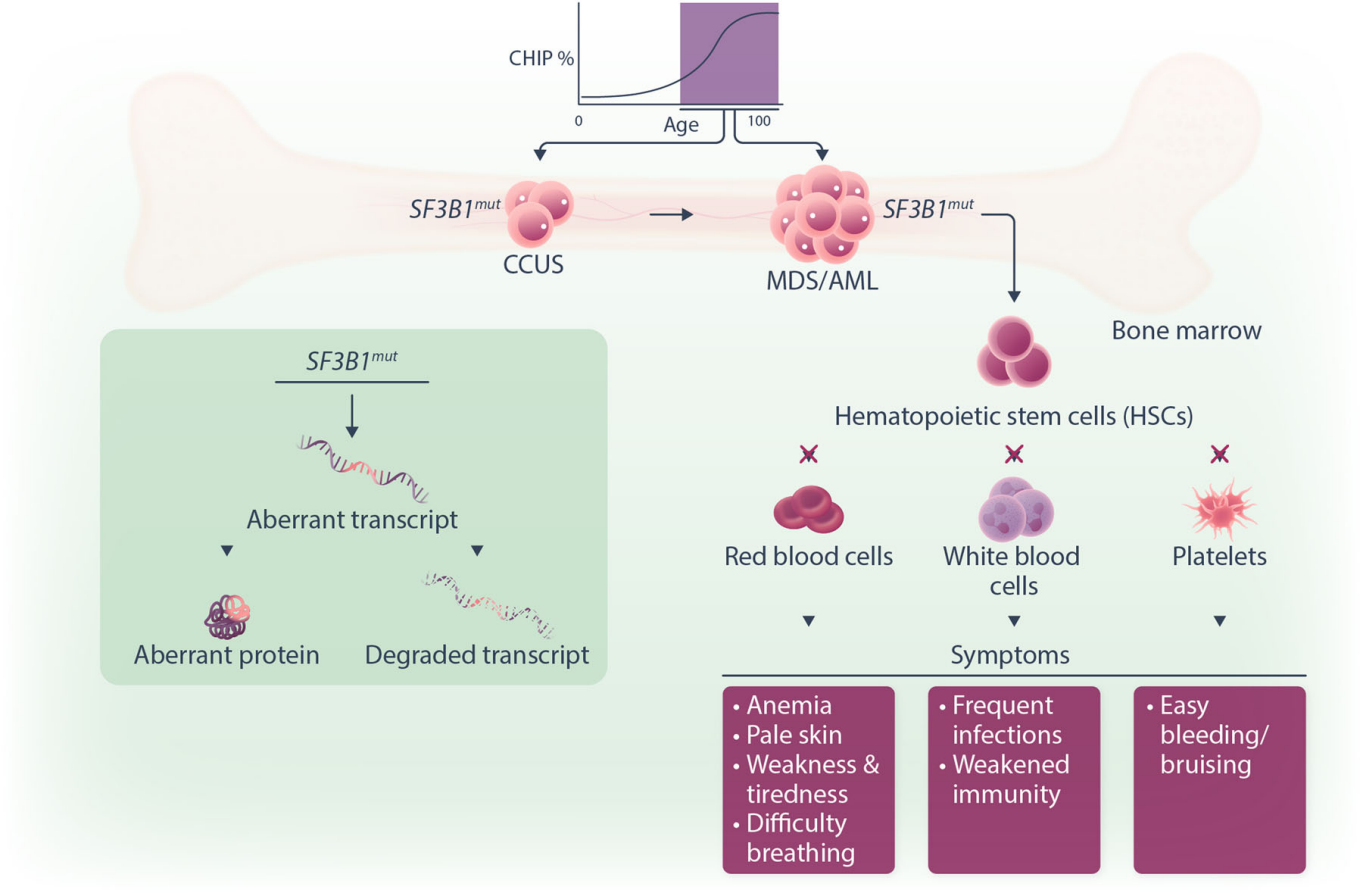
**Pathogenesis of MDS with** ***SF3B1*** **mutation.** *SF3B1* mutations occur in the hematopoietic stem cells (HSCs) that reside in the bone marrow. The SF3B1 protein is a core component of the U2 small nuclear ribonucleoprotein, involved in the recognition of the branch point sequence during the selection of the 3′ splice site in the RNA splicing process. Its mutations result in the misrecognition of 3′ splice sites that lead to aberrant mRNA transcripts either subjected to degradation or translated into aberrant proteins. *SF3B1* mutations are detected in Clonal Hematopoiesis of Indeterminate Potential,^41^ which may progress to Clonal Cytopenia of Undetermined Significance (CCUS). Eventually, MDS can manifest when substantial blast cells accumulate in the BM and contribute to BM dysplasia and cytopenia. Leukemic transformation leads to further dysplasia and abnormal white blood cell production. The resulting cytopenia contributes to a wide range of clinical sequelae.

MDS is a clonal bone marrow disorder, following the acquisition of genetic alterations in the hematopoietic stem cell compartment. Increasingly sensitive techniques have been utilized to trace the key genetic aberrations back to the phenotypic and functional hematopoietic stem cell (HSC) compartment, which is most well established in del(5q) MDS and *SF3B1*‐MDS.^36^, ^37^, ^38^, ^39^ These studies marked a pivotal turn in MDS biology, establishing MDS as a clonal heterogeneous disorder rooted in stem cells. Regarding *SF3B1*‐MDS, the mutations have been observed in the most primitive hematopoietic compartment. In contrast to AML, where the hematopoietic hierarchy is often perturbated, low risk MDS stem cells consistently preserved surface markers reminiscent of those found on healthy HSCs (CD34^+^CD38^+^CD45RA^−^CD90^+^).^38^, ^39^ These mutated MDS stem cells can replenish downstream myeloid and erythroid progenitors, establishing their hierarchical relationship. Subsequent studies have also demonstrated that *SF3B1‐*mutated progenitors may also be able to acquire self‐renewal capacities and propagate the disease.^40^ These findings align seamlessly with the recent observation that *SF3B1* mutations rank among the most prevalent genetic alterations identified in clonal hematopoiesis with an indeterminate potential,^41^ alongside *DNMT3A, TET2*, and *ASXL1.* ^2^, ^42^ CHIP is defined as the detection of at least one somatic mutation in the DNA of blood or bone marrow cells in the absence of a cytopenia or defined myeloid neoplasm.^43^ Patients with *SF3B1* clonal cytopenia of undetermined significance invariably develop MDS over time^28^ (Figure 1).

**Figure 2:**
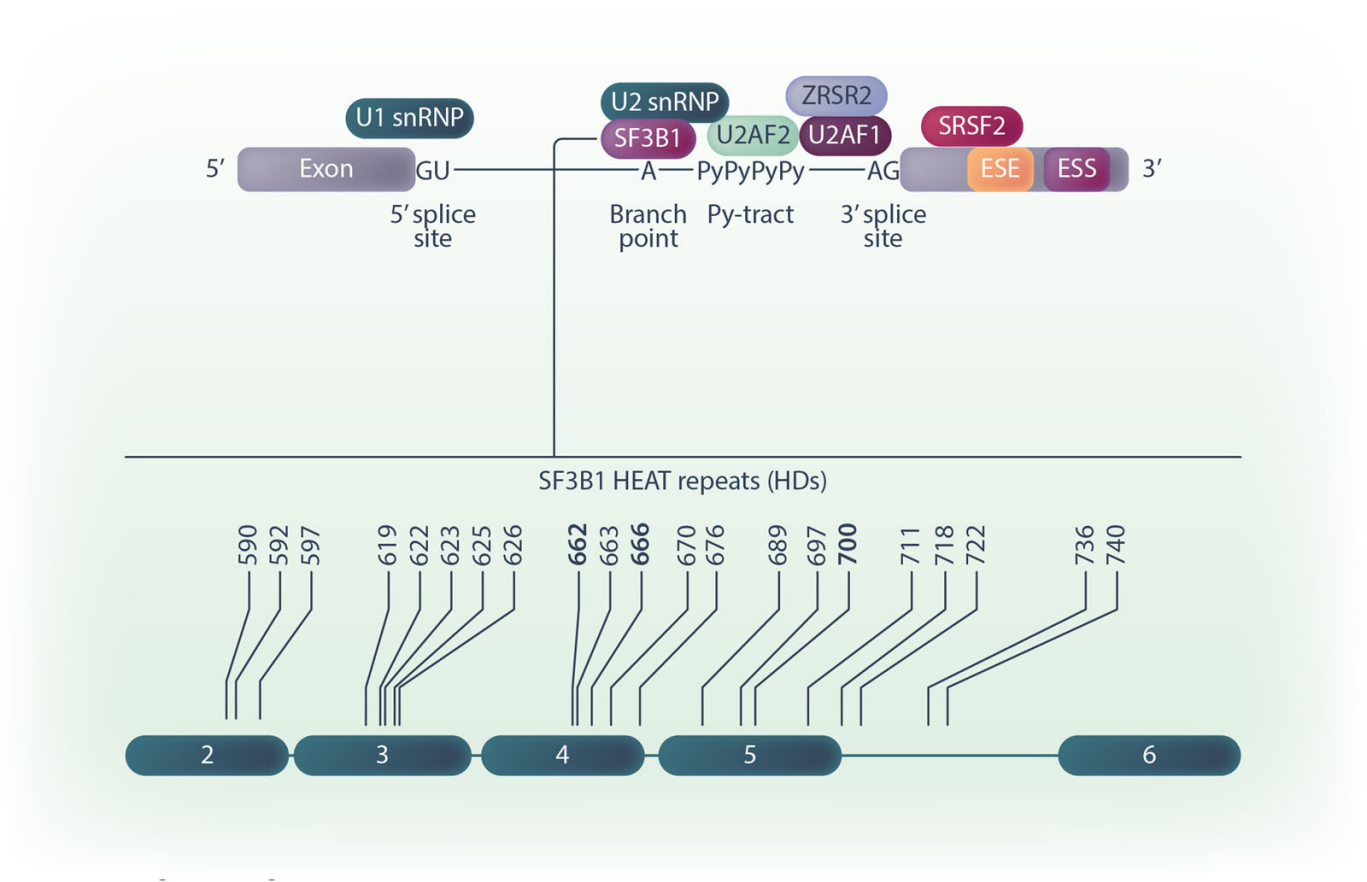
**Schematic of SF3B1 binding to the branch point sequence on pre‐mRNA and** ***SF3B1*** **most common mutations. Top schematic:** Representation of the major spliceosome bound to a pre‐mRNA. The U1 small nuclear ribonucleoprotein (snRNP) recognizes the 5′‐splice site; The U2 snRNP recognizes the 3′‐splice site at conserved sequence positions; SF3B1 binds to the branch point. **Bottom schematic**: *SF3B1* somatic mutations in HEAT Domain numbers 2–6. **Bold** = *SF3B1* hotspots in MDS.

Over the past decade, our understanding of the genetic landscape of MDS has improved greatly. Advances in next‐generation sequencing have revealed that the acquisition of recurrent mutations in a set of genes drives the initiation and progression of MDS.^29^ The most commonly mutated group of genes at the time of MDS diagnosis are the spliceosome genes: *SF3B1* (~25%–30%), *SRSF2* (~15%), *U2AF1* (~7%–11%), and *ZRSR2* (~5%) (Figure 2). These mutations often co‐exist with mutations in DNA methylation, chromatin modification such as *TET2, DNMT3A,* and *ASXL1,* and to a lesser extent with mutations in cell signaling genes and transcription factors.^44^ These mutations are major predictive factors with regard to clinical phenotype and in some cases prognosis. This has been an important step in understanding the molecular basis of MDS and has also provided clonal markers in patients with a normal karyotype. The identification of such driver mutations has provided unique genetic tools to track clonal diversity, for the first time enabling ‘fate mapping’ of genetic changes within MDS patients.

*SF3B1* is involved in an array of cellular processes, which contribute to the phenotype in *SF3B1*‐MDS, including the regulation of erythropoiesis, iron metabolism, inflammation, and R loop accumulation.^45^ *SF3B1, SRSF2, U2AF1,* and *ZRSR2* are key components of the splicing machinery conserved in eukaryotic organisms. These proteins are crucial for the splicing of introns and exons from pre‐mRNA, leading to the generation of different isoforms from a given gene. *SF3B1* mutations in MDS lead to the selection of a cryptic alternative 3’ splice site responsible for the generation of hundreds of mis‐splicing events, which are predicted to be either degraded by nonsense‐mediated decay or to produce aberrant proteins^46^, ^47^ (Figure 1).

*SF3B1* mutations are almost exclusively heterozygous missense mutations and typically occur in the HEAT domain with mutations in codon 700 (K700E) being the most frequent^48^, ^49^ (Figure 2). Attempts to model the disease in murine models have limitations due to the inter‐species disparities in the ribonucleotide sequence of the 3’ splice sites, emphasizing the importance of studying patient samples.^50^ Mis‐splicing of transcripts coding for *ABCB7, TMEM14C, MAP3K7, ERFE*, and *COASY* has been linked with ineffective erythroid differentiation and formation of ring sideroblasts.^51^, ^52^, ^53^, ^54^, ^55^ Mis‐splicing of mitotic regulators *BUBR1* and *CDC27* delays cell cycle G2/M transit and promotes CHK1 inhibitor sensitivity,^56^ whereas mis‐splicing of *IRAK4* leads to NF‐kB activation and inflammatory cytokine production.^57^ *SF3B1* mutations also promote the decay of transcripts encoding the protein phosphatase 2 A (PP2A) subunit PPP2R5A, increasing MYC and BCL2 phosphorylation hence promoting MYC protein stability and impairing apoptosis.^58^ Additionally, mutations in *SRSF2* and *U2AF1* display elevated R‐loops, replication stress, and activation of the ATR‐CHK1 pathway that represent very attractive therapeutic targets^59^ that require further attention. For instance, *SF3B1* mutations contribute to the loss of R‐loops with associated DNA replication stress^60^ although still controversial.^61^ In contrast, in SRSF2 and U2AF1 mutated MDS, R‐loop accumulation confers sensitivity to PARP1 inhibitors.^62^

**Figure 3:**
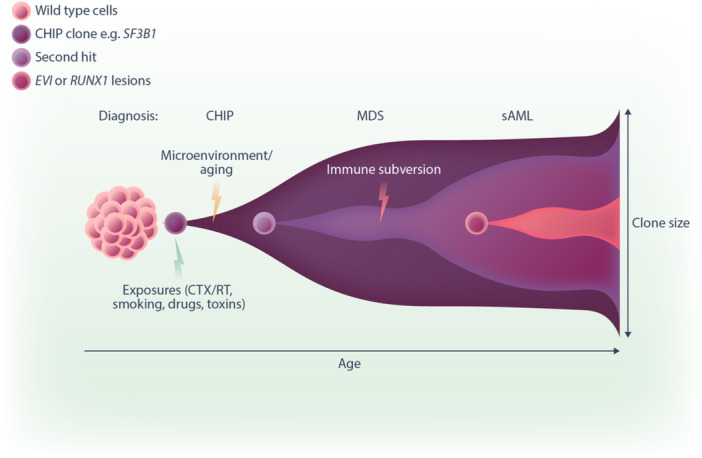
**Model of clonal evolution of** ***SF3B1**‐* **mutated clones.** CHIP, clonal hematopoiesis of indeterminate potential; CTX, chemotherapy; MDS, myelodysplastic syndromes/neoplasms; RT, radiotherapy; sAML, secondary AML.

Patients with *SF3B1*‐mutant MDS have a relatively good prognosis and are typically classified as low or very low risk MDS. However, transformation to AML is seen in 7% of cases.^63^, ^64^, ^65^ Additional mutations and a blast count of >5% are poor prognostic factors for transformation to AML.^64^ The presence of an *SF3B1* mutation designates it as AML with related myelodysplasia (AML‐MR), a subtype of AML with a dismal prognosis.^66^ In almost all cases, it co‐exists with other AML‐defining mutations.^63^ The two most common aberrations enriched at the AML stage in *SF3B1‐*mutated patients are *EVI1* (inversion or translocation of chromosome 3 – Inv3/t(3,3)) and *RUNX1* mutations^45^, ^63^, ^67^ (Figure 3).

## CURRENT THERAPEUTIC STRATEGIES IN THE GUIDELINES

### Low‐risk MDS—An unmet need

The only curative treatment for MDS is allogeneic hematopoietic cell transplantation (allo‐HCT). However, allo‐HCT carries a significant risk of transplant related mortality (TRM) and relapse, with a TRM between 15% and 50%^68^ with less than 50% of patients achieving long term cure.^69^ For fit patients with LR‐MDS, including *SF3B1*‐mutated MDS, there are ongoing trials regarding the potential benefit of early transplantation,^70^, ^71^ but for most patients, options remain supportive and non‐curative.

Despite LR‐MDS comprising three‐quarters of all MDS cases, a recent study found that only 6% of clinical trials in MDS focused on evaluating treatments for LR‐MDS patients, in contrast to the attention given to its high‐risk counterpart.^72^ The reasons for this are likely manifold, including the difficulty in recruiting multi‐morbid patients into clinical trials, an older patient population, the paucity of clinically meaningful endpoints, and the lack of perceived urgency to improve treatments in a disease that does not rapidly transform to acute leukemia.

**Figure 4:**
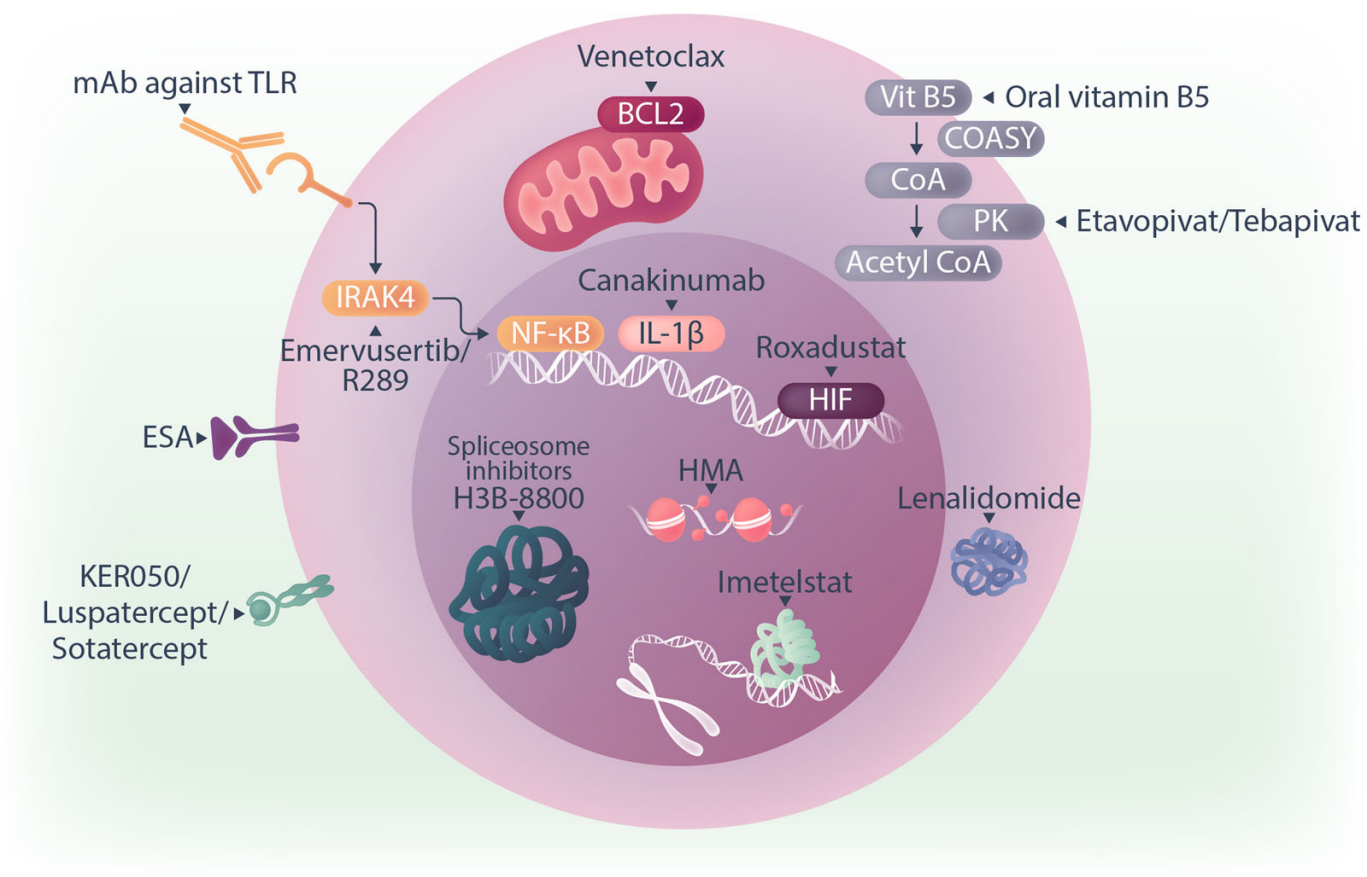
**Targeted therapies under exploration and in the clinic for MDS**. BCL2, B‐cell leukemia/lymphoma 2 protein; CoA, co‐enzyme A; COASY, co‐enzyme A synthase; ESA, erythropoietin stimulating agent; HIF, hypoxia inducible factor; HMA, hypomethylating agents; IL‐1b, interleukin‐1b; IRAK4, interleukin‐1 receptor‐associated kinase 4; NFkB, nuclear factor kappa‐light‐chain‐enhancer of activated B cells; mAB, monoclonal antibody; PK, pyruvate kinase; TLR, Toll‐like receptor; Vit B5, vitamin B_5_.

In LR‐MDS, the mainstay of management is the maintenance and improvement of quality of life, focusing on supporting the bone marrow function and ameliorating cytopenias. Anemia is present in approximately 85% of MDS patients at the time of diagnosis^73^ and is a major cause of morbidity and mortality.^73^, ^74^, ^75^, ^76^ At least 50% of patients with MDS become red cell transfusion‐dependent.^77^ Red cell transfusion increases the risk of transfusion reactions, iron overload, and antibody formations, as well as worsening cardiopulmonary function.^78^ Additionally, it may contribute to impaired cognitive function.^79^, ^80^ All of these features impact a patient's quality of life while being burdensome for both patients and healthcare resources,^81^, ^82^ demonstrating the unmet clinical need for new therapeutics. The current and putative treatments are illustrated in Figure 4 and Table 2.

**Table 2:** Current and emerging treatments for *SF3B1‐*mutated MDS.

Treatment	Clinical trial number or reference	Target/mechanism of action	License in Europe/UK/USA
ESAs		Stimulating the transcription of genes regulating the late maturation and proliferation of erythrocytes in the bone marrow.	1st line treatment of all LR‐MDS in Europe and in the UK. Not FDA approved for this indication but widely used.
– Epoeitin beta	NCT02428686^2^		
	NCT02145026^4^		
– Epoeitin alfa	NCT01381809^3^		
– +/− GCSF	NCT00003138^3^	GCSF may synergise with ESAs to increase differentiation and mobilization of progenitor cells from the bone marrow while also preventing apoptosis of progenitors.	
Ligand traps		A receptor fusion protein that binds to TGF‐β and acts as a ligand trap to neutralize negative regulators of late‐stage erythropoiesis.	Luspatercept for first‐line treatment of all LR‐MDS and intermediate risk if *SF3B1* mut in Europe. In the UK, only as second‐line treatment. FDA approved for anemia failing an erythropoiesis stimulating agent and requiring two or more RBC units over 8 weeks in adult patients with very low‐ to intermediate‐risk myelodysplastic syndromes with ring‐siderobalsts.
– Luspatercept	NCT03682536^3^		
	NCT02631070^3^		

– KER‐050	NCT04419649^2^	No license for KER‐050.
Iron chelation		Binds iron to mitigate iron overload.	For all MDS patients who require frequent red blood cell transfusions and/or have evidence of chronic iron overload. In UK, deferasirox is only licensed second line (after desferrioxamine).
	NCT01868477^2^		
	NCT03387475^2^		
	NCT00469560^3^		All are FDA‐approved and approved in Europe.
	NCT00117507^4^		
Hypomethylating agents		Modification of epigenetic regulation of genes by inhibition of DNA methylation.	Azacitidine is not licensed in the UK or Europe for treatment for LR‐MDS outside of a trial. Decitabine is licensed after failure of first‐ or second‐line treatment in Europe.
– Azacitidine	NCT01048034^2^		
	NCT00897130^2^		Both are FDA‐approved for all MDS subtypes.
	NCT01338337^2^		
	NCT01566695^3^		
– Decitibine	NCT03502668^1/2^		
	NCT00619099^2^		
	NCT01720225^2^		
Stem cell transplantation	NCT02757989^n/a^	Allows high doses of chemotherapy to be given with stem cell rescue, as well as immune modulatory of graft‐versus‐leukemia effect.	Can be considered if there are poor prognostic factors
Telomerase inhibitors		Selectively target cells with increased telomerase activity, such as MDS cells, where telomerase may drive the replicative immortality and dominance in the bone marrow of the malignant progenitor cell clone.	None in Europe/UK. FDA‐approved in June 2024 for adults with low‐ to intermediate‐1 risk MDS and transfusion‐dependent anemia requiring four or more RBC units over 8 weeks who have not responded to, have lost response to, or are not eligible for ESAs.
– Imetelstat	NCT02598661^2/3^		
Spliceosome modulators		Bind to the spliceosome and inhibit splicing.	None
– H3B‐8800	NCT02841540^1^	Inhibitors of PRMT inhibit dimethylation of arginine and reduce splicing fidelity and result in the preferential killing of splicing factor‐mutated cells.	
– E7107	NCT00499499^1 (suspended)^		
– PRMT5 inhibitors:			
– PRT543	NCT03886831^1^		
– JNJ‐64619178	NCT03573310^1^		
Immune modulators:			None
– Tomaralimab	NCT02363491^1/2^	Monoclonal antibody against a TLR to moderate immune escape.	
– Emavusertib	NCT04278768^2a^	Dual IRAK4 (TLR mediator) and FLT3 inhibitor.	
– R289	NCT05308264^1b^	An IRAK1/4 inhibitor	
– Canakinumab	NCT04239157^2^	Anti‐IL‐1β Human Monoclonal Antibody	
Vitamin B_5_	Mian, S.A., et al. Sci Transl Med, 2023	Increases the substrate for the production of succinyl co‐A and therefore heme production	None
Pyruvate kinase activators		Targeting glycolysis in RBC metabolism	None
– Tebapivat	NCT05490446^2a/b^		
– Etavopivat	NCT05568225		
Erythroferrone antagonists	Bondu, S., et al. Sci Transl Med, 2019	Targeting low hepcidin levels and therefore decreasing iron overload	None
MYC and BCL2 inhibition		Target the MYC and BCL2 apoptotic programs	None
– PP2A inhibitors	Liu, Z., et al. Cancer Discov, 2020		
	Yeh, E., et al. Nat Cell Biol, 2004		
– Venetoclax	NCT02966782^1b^		

*Note*: License, target/mechanism of action, and trial were included when available.

Abbreviations: ESAs, erythropoiesis‐stimulating agents; GCSF, granulocyte colony stimulating factor; IL, interleukin; IRAK, interleukin‐1 receptor‐associated kinase; PRMT, protein arginine methyltransferases; RBC, red blood cell; Superscript above the trial, the phase of the trial; TGF, transforming growth factor; TLR, Toll‐like receptors.

### Supportive care

At the beginning of the patient journey, many patients with *SF3B1*‐mutated MDS require no supportive treatments and are actively monitored with regular blood tests and clinical review. This entails regular monitoring of patients to assess symptoms and hematological indices, as there is no high‐quality evidence supporting the advantage of earlier intervention.^83^ However, when the anemia reaches symptomatic levels, approved treatment options are broadly divided between erythropoiesis stimulating agents (ESAs), transfusions, and most recently, Luspatercept.^84^

#### ESAs

ESAs work by stimulating the transcription of genes regulating the late maturation and proliferation of erythrocytes in the bone marrow in a manner similar to endogenous erythropoietin.^85^, ^86^ The Nordic scoring system was developed by Hellstrom‐Lindberg and colleagues to predict response to ESAs in MDS patients.^87^ It is comprised of two weighted elements; a patient's endogenous erythropoietin levels and transfusion dependency status, to classify patients into high, medium, or low probability of ESA response.^88^, ^89^ In a randomized phase 3 study, 147 low‐risk MDS patients with hemoglobin (Hb) level of ≤10 g/dL, EPO level of ≤500 mU/mL, and low transfusion load were randomized to receive ESA or placebo.^76^ Following placebo or subcutaneous administration of 500 µg DAR‐alpha every 3 weeks for 24 weeks, the frequency of transfusion was significantly higher in the placebo arm (59.2 vs. 36.1% and *p* = 0.008), while the proportion achieving an erythroid response (defined as ⩾1.5 g/dL increase from the baseline in hemoglobin with a mean rise of ⩾1.5 g/dL for 8 weeks) was significantly higher in the ESA arm (14.7 vs. 0% and *p* = 0.016). A similar study with EPO‐alpha showed that the recovery of anemia was significantly higher in the EPO‐alpha arm (31.8 vs. 4.4% and *p* = 0.001).^90^

As one of the hallmarks of *SF3B1*‐mutated MDS is anemia caused by marked ineffective maturation of erythroblasts in the bone marrow, this commonly results in a higher endogenous EPO level, rendering patients with *SF3B1* mutation less responsive to ESA.^91^, ^92^ Conversely, the MDS Clinical Research Consortium compared ESA response rates in patients with wild type versus *SF3B1‐*mutated MDS. 14/40 (35%) of erythroid‐stimulating agent‐treated patients with *SF3B1‐*mutated MDS experienced response versus 9/56 (16%) wild‐type patients (*p* = 0.032)^93^ showing a preferential response of *SF3B1‐*mutated patients to ESAs. Response rates are still low, and the duration of response remains short. Most responses to ESAs occur within 3 months of treatment and have a median duration of about 15–18 months^94^; this reversion to transfusion dependence is even more pronounced in patients with ring‐sideroblasts (95% of whom harbor an *SF3B1 mutation*).^73^, ^95^

Granulocyte colony stimulating factor (G‐CSF) may synergize with ESAs to increase differentiation and mobilization of progenitor cells from the bone marrow while also preventing apoptosis of progenitors.^96^, ^97^ This effect might be more pronounced in LR‐MDS patients with ring‐sideroblasts.^94^, ^97^ The role of G‐CSF on blast proliferation was assessed, and to date, there is no demonstration that it increases the blast percentage and no increase in the risk of transformation into AML.^98^ Thus, the addition of G‐CSF to ESAs is in several treatment algorithms.^99^, ^100^

#### Luspatercept

Luspatercept is a receptor fusion protein that binds to TGF‐β and acts as a ligand trap to neutralize negative regulators of late‐stage erythropoiesis, releasing the differentiation block seen in MDS patients.^101^, ^102^ It was approved by the FDA as frontline therapy for lower‐risk MDS and transfusion‐dependent anemia in August 2023. Its initial approval was secured following the MEDALIST trial, which was a phase 3 randomized study of luspatercept versus placebo for the treatment of anemia in patients with lower‐risk MDS, with ring sideroblasts, of whom 93% of participants harbored an *SF3B1* mutation and had failed ESA or were unsuitable for ESA therapy. Patients with very low, low, and intermediate risk groups according to IPSS‐R were randomized 2:1 to luspatercept and placebo arms. Luspatercept significantly improved 8‐week transfusion independence within 24 weeks of trial enrollment (38 vs. 13% *p* < 0.001), with no difference in AML transformation. Following MEDALIST, the COMMANDS study compared first‐line Luspatercept versus ESA in patients with lower‐risk MDS and red cell transfusion dependence, regardless of the presence of ring sideroblasts or *SF3B1* mutations, meeting its primary endpoint, demonstrating superior achievement of transfusion independence (TI) in the Luspatercept versus ESA arm (59% v 31%) with a mean increase in hemoglobin of 1.5 g by 24 weeks (*p* < 0.0001).^103^ As part of the COMMANDS trial mutational analysis of the patients and subgroup results, mutational analysis of the patients and subgroup results showed superior results with Luspatercept in patients with SF3B1 mutations,^103^, ^104^ consistent with data observed in the MEDALIST trial.^74^ While the precise cause remains uncertain, it has been suggested that the observed phenomenon may be attributed to the downregulation of spliceosome pathways by Luspatercept,^74^, ^105^ along with the downregulation of pro‐inflammatory signals in the bone marrow that may contribute to the restoration of a normal hematopoietic environment.^74^ Other ligand traps, such as KER‐050, are also under investigation, with clinical trials (KER‐050‐D301) currently evaluating its efficacy, specifically within *SF3B1*‐MDS.

#### Red cell transfusions and iron chelation

Despite recent advances, the majority of patients with *SF3B1‐*mutated MDS become red cell transfusion‐dependent. The mean number of red blood cell units required is 2 every 4 weeks.^106^ Iron overload is a side effect of frequent transfusions, which can result in cardiac and liver failure, an increase in the risk of infection, and mortality.^80^

Iron overload is prevalent in MDS, especially in cases with SF3B1 mutations. This is largely due to red blood cell transfusion, and it is accentuated by the downregulation of hepcidin, a protein hormone crucial for iron homeostasis, which is particularly pronounced in MDS patients. The lowest hepcidin levels are observed in patients with ring sideroblasts.^107^ Patients may develop systemic iron overload even before becoming transfusion‐dependent.^108^ Upregulation of Erythroferrone (ERFE), a protein that is produced in response to increased EPO production^52^ together with other causes of ineffective erythropoiesis in patients with *SF3B1* mutations, causes a decrease in hepcidin and subsequent iron retention and overload.^109^, ^110^, ^111^, ^112^

While some side effects from RBC transfusion can only be mitigated with careful and judicious transfusion regimens (such as circulatory overload or antibody production), iron overload can be effectively controlled with chelating agents. The TELESTO trial^113^ was a phase 2 randomized placebo‐controlled study of 225 patients investigating deferasirox in LR‐MDS patients with serum ferritin >1000 ng/mL and transfusion history of 15–75 red cell units. Iron chelation was associated with a 36.4% risk reduction in event‐free survival, and interestingly up to 39% of patients on iron chelation had improvement in their hematological parameters.^113^ This is in line with previous non‐randomized studies, which showed an improvement in hemoglobin in patients receiving iron chelation, even to the point of becoming transfusion independent.^84^, ^114^, ^115^, ^116^, ^117^ In line with these data, another study, evaluating the impact of iron chelation therapy on the overall survival of transfusion‐dependent lower‐risk MDS patients, reported longer median overall survival from the time of transfusion dependence in patients receiving chelation (5.2 vs. 2.1 years, *p* < .001), adjusting for age, comorbidity, and other patient‐related factors.^118^, ^119^ In order to evaluate the impact of iron chelation therapy on achieving transfusion independence in patients with low‐risk MDS, the clinical trial NCT03387475 was conducted in France between 2018 and 2023. The preliminary results presented at the American Society of Hematology annual congress in 2024 (https://doi.org/10.1182/blood-2024-198104 NOT YET AVAILABLE for referencing) showed that the primary objective, which was to achieve transfusion independence at 12 months, was met in 47.4% (CI‐95%: 31.0%; 64.2%) of the patients daily treated with 3.5 mg/kg of deferasirox.

Furthermore, transfusion dependency and iron overload negatively impact patient's health‐related quality of life (HRQoL)^82^ and should be routinely assessed with tools like The Quality of Life in Myelodysplasia Scale (QUALMS) or The Functional Assessment of Cancer Therapy‐Anemia (FACT‐An) questionaries that measure items such as physical and emotional burden.^52^, ^120^, ^121^

### Alternative therapeutics

#### Hypomethylating Agents

Regarding spliceosome mutations specifically, a small cohort study in AML showed that a venetoclax‐HMA regime has similar OS across mutated and WT patients. This is notable in AML as spliceosome mutations carry a poor prognosis, unlike in MDS.^122^ In another cohort study, 133 MDS patients were examined for somatic mutations in *SF3B1, U2AF1,* and *SRSF2,* and 59 out of 133 patients received treatment with an HMA.^123^ The treatment indications for the HMA were MDS with IPSS intermediate‐1 with anemia and no response to EPO or with another cytopenia, intermediate‐2, or high risk. In this study, HMAs showed no difference in treatment response and overall survival for WT or spliceosome‐mutated MDS, emphasizing the unmet need for these patients. Currently, a Phase 1‐2, multicenter, open‐label study of various ASTX727 LD (decitabine/cetirizine) doses is open to assess the safety and pharmacodynamics in low‐risk MDS (NCT03502668).

#### Stem cell transplantation

While allogeneic stem cell transplantation is the only curative option for myelodysplastic syndrome (MDS), its use in low‐risk MDS (LR‐MDS) is generally avoided due to the high morbidity and mortality associated with the procedure.^124^ However, a study describing the outcomes of 438 patients with lower‐risk MDS after failure to HMAs has demonstrated good outcomes (median survival of 39 months vs. 10 months vs. 28 months for transplant vs. no further therapy vs. conventional therapy, respectively).^125^ Using stochastic modeling, there have been two studies reviewing the data on allogeneic stem cell transplant, and even with reduced intensity regimes, these showed that the risk outweighs the benefit in LR‐MDS patients in the absence of high‐risk features.^126^, ^127^ There is a currently active open‐label clinical trial for allogeneic transplant in patients with LR‐MDS provided they have at least one feature of high‐risk disease (NCT02757989). Other immune suppressive treatments also carry the risk of infection and are generally limited to people with hypoplastic MDS who share features with aplastic anemia.^128^

## RECENTLY PUBLISHED MDS TRIALS

### Imetelstat

Telomeres, composed of protein and non‐coding DNA, reside at chromosome ends, safeguarding the genome from degradation. Telomerase is the enzyme responsible for maintaining telomere length. Imetelstat, a telomerase enzyme inhibitor, has recently been FDA‐approved for patients with transfusion‐dependent LR‐MDS who are ineligible or refractory to ESA. Patients with MDS often have short telomeres,^129^ although telomerase activity and expression of human telomerase reverse transcription (hTERT; a key catalytic subunit of telomerase) are often increased in MDS cells and may drive the replicative immortality and dominance in the bone marrow of the malignant progenitor cell clone.^130^, ^131^ By selectively targeting cells with increased telomerase activity, imetelstat may selectively induce apoptosis of the malignant clone. IMerge, a phase 3 study of imetelstat,^132^ recruited 178 patients in 118 sites with ESA‐relapsed, ESA‐refractory, or ESA‐ineligible LR‐MDS. 91 (77%) of 118 patients had discontinued treatment by data cutoff in the imetelstat group versus 45 (75%) in the placebo group. In the imetelstat group, 40% of patients were transfusion independent at 8 weeks versus 15% in the placebo group. 17.8% of imetelstat‐treated patients and 1.7% of patients on placebo achieved one year transfusion independence. No treatment‐related deaths were reported although the most common treatment‐related adverse events of thrombocytopenia and neutropenia were more common in the imetelstat group. The study is the first to show a reduction in the variant allele frequency (VAF) of the most commonly mutated genes in MDS: *SF3B1, TET2, DNMT3*A, and *ASXL1*. Out of the 18 one year responders to imetelstat for whom mutational data were available, 13 (72.2%) achieved greater than or equal to 50% *SF3B1* VAF reduction, including 7 patients with an undetectable VAF,^132^ showing the potential for this drug to not just provide clinical benefit, but disease modification.

### Spliceosome modulators

The discovery of ubiquitous splicing mutations in MDS as mutually exclusive somatic mutations has led to an interest in splicing inhibitors and modulators. One of the first discovered pladienolide binds to the spliceosome and inhibits splicing, which was thought to cause synthetic lethality in tumor cells.^133^ The first splicing modulator to enter the clinic was pladienolide's semisynthetic derivative E7107; human trials against solid tumors were halted owing to dose‐limiting toxicity and showed little or no clinical benefit.^134^, ^135^

Spliceostatin A,^136^ and Jerantinine A have demonstrated synthetic lethality causing tumor cell death,^137^ but neither of these have made it to clinical trial due to difficulties in synthesis and unexpected toxicities in animal models.

A synthetic pladienolide derivative H3B‐8800 was the first compound tested in the spliceosome mutated on hematopoietic cell lines, including *SF3B1*, *SRSF2,* and *U2AF1.* ^138^ In a phase 1 clinical trial, it was tested in 84 patients with myeloid cancers (42 with HR‐MDS or LR‐MDS; 88% with spliceosome mutations of interest) and only 14% of patients experienced reduced transfusion requirement and marrow responses while changes in mutation burden were not seen.^139^, ^140^ Therefore, despite spliceosome modulators holding great promise, their use has so far been limited by excessive toxicity and lack of efficacy.

Another approach for targeting splicing factor mutant cells is the use of inhibitors of the protein arginine methyltransferase (PRMT). In pre‐clinical studies, inhibitors of these enzymes were shown to reduce splicing and resulted in the preferential killing of splicing factor mutant leukemia cells compared to their wild‐type counterpart.^141^ Phase 1/2 studies investigating the safety and clinical activity of the PRMT5 inhibitors PRT543 in patients with hematological malignancies (NCT03886831) and JNJ‐64619178 in patients with low‐risk MDS (NCT03573310) are recruiting.

### Inflammatory pathway inhibitors

It is well known that inflammatory signaling dysregulation is key in the pathogenesis of MDS.^142^, ^143^ To escape recognition by immune cells, cancer cells have evolved to lose their immunogenicity and that of their environs.^144^ An example of such immune escape is Toll‐like receptors (TLRs), which normally mediate innate immune response and are over‐expressed in MDS cells. Their aberrant activation contributes to ineffective hematopoiesis.^145^

Pollyea et al. reviewed the relationship between splicing factor‐mutated MDS and innate immunity contributing to disease pathogenesis, discussing the fact that mutations in *SF3B1*, *U2AF1*, and *SRSF2* enhance NFκB activity and LPS‐induced inflammatory cytokine production in cell lines, primary patient samples, and murine models.^146^

*SF3B1‐*mutated cells specifically showed that when MAP3K7, a protein known to be mis‐spliced in *SF3B1* cells, had a TLR agonist added to them, this resulted in an increase in the inflammatory NF‐κB pathway.^147^ A similar study investigated macrophages, patient‐derived cell lines, and both mouse and human bone marrow cells, revealing significant changes in pre‐mRNA splicing and gene expression in hematopoietic progenitor cells of MDS patients with *SF3B1* mutations. The study demonstrated upregulation of several pro‐inflammatory signaling pathways, including enhanced NF‐κB activity and increased LPS‐induced production of inflammatory cytokines.^148^

A phase I/II clinical trial of a monoclonal antibody against TLR2, in transfusion‐dependent patients with low‐ or intermediate‐risk MDS in whom HMA therapy had failed, was done and showed an ORR of 50%, which suggests that targeting TLR2 may improve erythropoiesis in this cohort.^149^

Interleukin‐1 receptor‐associated kinase 4 (IRAK4) is a key mediator of TLR and interleukin‐1 receptor‐induced NF‐κB signaling pathway activation and triggers inflammatory responses and survival mechanisms in cancer cells.^150^ It has been shown that *U2AF1* and *SF3B1* mutations lead to aberrant splicing of *IRAK4*, which results in a longer isoform, which significantly increases the activation of the NF‐κB pathway, and furthermore, the inhibition of this isoform rescues cell differentiation.^57^, ^151^ As IRAK4 signaling was also thought to be a mechanism of adaptive resistance in the setting of FLT3‐mutant AML,^152^ a phase I/II trial of Emavusertib, a novel oral dual IRAK4 and FLT3 inhibitor, was undertaken in patients with R/R MDS/AML. Preliminary results from this trial showed that Emavusertib as a single agent or in combination with 5‐aza or venetoclax has efficacy in patients with *SF3B1*, *U2AF1*, or *FLT3* mutations. Importantly, in the seven patients with MDS with spliceosome mutations, 57% reached marrow complete remission, including one with red blood cell transfusion independence.^153^, ^154^ R289 is an IRAK1/4 inhibitor, currently undergoing phase 1b clinical trial in the United States (C‐906289‐002). Similarly, Canakinumab, which inhibits IL‐1b receptor binding, is in a phase‐2 trial for LR‐MDS (NCT04239157).

## THE FUTURE: THERAPEUTIC TARGETING AND RELEVANCE BEYOND *SF3B1‐*MUTATED MDS

*SF3B1‐*mutated MDS has unique therapeutic vulnerabilities ripe for exploitation by existing treatments. *SF3B1*mut often undergoes aberrant activation of the DNA damage response due to mis‐spliced proteins, thus existing DNA damaging agents (e.g., etoposide) or synthetic lethal small molecule inhibitors (e.g., PARP inhibitors) could preferentially target tumor cells carrying *SF3B1* mutation.^45^, ^155^ The unique pathophysiology of *SF3B1* also makes it an exciting target for potential novel treatments that can inform further work for cancer therapeutics.

### Vitamin B_5_

Coenzyme A synthase (COASY) is a core bifunctional enzyme that catalyzes from vitamin B5, the fourth and fifth sequential steps of the CoA biosynthetic pathway, and is mis‐spliced in *SF3B1‐*mutated cell lines and patient samples. This mis‐splicing leads to the loss of 50% of COASY protein expression, which plays a critical role during erythropoiesis through the production of succinyl‐CoA.^53^, ^156^ The supplementation of vitamin B5 to saturate the available COASY or the addition of succinyl‐coA rescued patient cells' ineffective erythropoiesis *ex‐vivo* ^53^ These findings open up new perspectives for treatments of *SF3B1*‐MDS that aim at improving patients' quality of life via combinatory treatment, hereby with the strong rational that vitamin B5 supplements^157^ could improve *SF3B1‐*MDS erythropoiesis.^157^ Interestingly, the opposite approach could be envisaged by depleting vitamin B5 from patients' diets to induce catastrophic synthetic lethality in *SF3B1* malignant clones.

### Pyruvate kinase

The impaired metabolism in erythrocytes in MDS has long been a subject in question, due to the similar phenotype that it has to congenital anemias.^158^ Glycolysis is the prominent metabolic cascade in RBCs and is currently targeted with pyruvate kinase (PK) activators in clinical trials in rare anemias^159^, ^160^ as well as shows high activity in MDS patient samples. A phase 2a/2b study is currently underway with Tebapivat, a potent PK activator, in LR‐MDS (NCT05490446). Similarly, Etavopivat, a PK activator under investigation for sickle‐cell disease, is being investigated in the phase 2 study FORTITUDE (NCT05568225) evaluating hematologic improvements in patients with very low‐, low‐, and intermediate‐risk MDS.

### MYC and BCL‐2

Using a transcriptional molecular interaction network, researchers found that tumors harboring *SF3B1* K700E mutations activate the MYC transcriptional oncogenic program.^161^ *PP2R5AA* (part of the PP2A complex) that drives MYC degradation is mis‐spliced by *SF3B1* and leads to nonsense mediated decay (NMD) of PP2A's transcript.^162^ Remarkably, *SF3B1*‐mutated cells are more sensitive to activators of PP2A (such as Fingolimod, FTY720, an immune modulator already used in clinical practice for multiple sclerosis^163^), suggesting the importance of MYC program activation in *SF3B1* malignant clones and advocating for it to be targeted.

*BCL2* is a member of the BH3 family gene and is a well‐known anti‐apoptotic gene upregulated in many cancers.^164^ BCL2 phosphorylation on the residue serine 70 (S70) is necessary for its anti‐apoptotic function,^165^, ^166^ and this is regulated by PP2A. *SF3B1* mutations promote the decay of transcripts encoding *PPP2R5A*, increasing BCL2 S70 phosphorylation. Genetic PPP2R5A restoration or pharmacologic PP2A activation impaired *SF3B1*‐mutant tumorigenesis elucidating a therapeutic approach to aberrant splicing by mutant *SF3B1*. S70‐phosphorylated BCL2 is increased in *SF3B1* mutated cells, which therefore was less apoptotic, and this was rescued by PPP2R5A restoration.^58^ BCL‐2 inhibitors are also in widespread use in other hematological malignancies, and a specific PP2A inhibitor, LB‐100, is currently in use for solid oncology tumors, undergoing a phase 1b/2 clinical trial (NCT03886662).

A study that mapped the determinants of AML response to different drugs by using CRISPR editing of different genetic mutations in an AML cell line established a functional link between splicing modulation and therapeutic efficacy of BCL2 inhibition in AML.^167^ The screens showed that the loss of RNA splicing factors preferentially enhances response to the BCL2 inhibitor. Inhibition of the RNA binding protein (RBM10) and splicing kinase families (CLKs and DYRK) combined with BCL2 inhibition led to mis‐splicing and inactivation of apoptotic inhibitors, which are implicated in BCL2 inhibitor resistance. Similarly, the inactivation of several serine/arginine‐rich proteins (SRSF2, SRSF3, SRSF8, and SRSF11) sensitized AML cells to BCL2 inhibition.^167^

A phase 1 trial of azacitidine with venetoclax in relapsed/refractory HR‐MDS with enrolled 12 patients. A response was seen in 8 patients, with 3 patients achieving transfusion independence,^168^ the phase 1b study conducted subsequently, included 6 out of 44 patients with SRSF2 mutations, 4 with SF3B1, and 6 with LR‐MDS. Complete remission was observed in 3 patients, marrow remission in 14 patients, 16 patients achieved transfusion independence, and a further 6 patients achieved hematological improvement.^169^

### Erythroferrone

Erythroferrone (ERFE) is a member of the C1q–tumor necrosis factor–related family of proteins and has been described as a major erythroid regulator of hepcidin.^170^ Mis‐splicing of *ERFE* by the mutant *SF3B1* generates an alternative transcript that leads to ERFE protein over‐expression^52^ that has been shown to lead to decreased hepcidin expression and increased iron overload.^171^, ^172^, ^173^ Murine models of thalassemia and hereditary hemochromatosis have demonstrated reduced iron loading with hepcidin agonists.^174^, ^175^ The ongoing TRANCEND study (NCT03802201), investigating the hepcidin mimetic PTG‐300 in Thalassemia, has shown promising preliminary phase 2 study results. Additionally, a recent study reported that SLN124, a GalNAc conjugated 19‐mer siRNA targeting *TMPRSS6*, effectively reduces plasma iron levels and increases hepcidin in healthy volunteers.^176^ A phase 1 clinical trial of SLN124 was conducted in patients with thalassemia or low‐risk MDS (NCT04718844). Antibodies against erythroferrone have so far only been tested on murine thalassemia models.^177^ Conversely, studies have linked higher hepcidin levels with higher‐risk MDS and have shown that excessive hepcidin levels lead to anemia, warranting caution and more studies in this area.^112^, ^178^

## CONCLUSIONS

*SF3B1‐*mutated MDS has emerged as a distinct entity, evidenced by unique laboratory and clinical features. Increasing data also supports a heightened response to specific therapeutic agents such as Luspatercept to rescue anemia, the most common and debilitating cytopenia in this patient group. However, it is still the case that the majority of patients quickly become red cell transfusion‐dependent, highlighting this area of unmet clinical need.

Growing evidence highlight the role of immune and stromal dysregulation; however, their contributions to the pathogenesis and defective erythropoiesis of MDS remain largely unexplored despite their potential key impact on patients' management and targeted therapies. The International Integrative Innovative Immunology for MDS (i4MDS) consortium is tackling the difficult question of establishing immunological profiling alongside molecular profiling, in order to shed light on the role of the immune microenvironment in MDS pathogenesis and progression.^179^ It is also known that the stromal microenvironment is reprogrammed by MDS cells^180^ or can induce bone marrow failure when dysregulated^181^, ^182^, ^183^ while also potentially nurturing clonal expansion through pro‐inflammatory stimuli.^184^ These are pending questions that require international efforts to be answered.

To conclude, recent experimental approaches have begun to elucidate the mechanism behind SF3B1 mutant‐mediated dyserythropoiesis, paving the way for the development of targeted therapies in this arena, where specific therapeutic vulnerabilities may be exploited, to provide a precision medicine that is not only effective but accessible and well tolerated.

## AUTHOR CONTRIBUTIONS

S.B., O.C., and K.R.P. designed, wrote, and reviewed the manuscript. O.C. and K.R.P. supervised the work.

## CONFLICT OF INTEREST STATEMENT

O.C. is a consultant for Sobi; and speaker at events for Novartis and Jazz.

## FUNDING

O.C received funding from MRC award MR/T005211/1. S.B. and K.R.P. received funding from the HARP/Wellcome trust program (223500/Z/21/Z) and the Barts Charity (G‐002167).

## Data Availability

Data sharing not applicable to this article as no data sets were generated or analyzed during the current study.
